# Electroclinical features and phenotypic differences in adenylosuccinate lyase deficiency: Long‐term follow‐up of seven patients from four families and appraisal of the literature

**DOI:** 10.1002/epi4.12837

**Published:** 2023-11-27

**Authors:** Gianni Cutillo, Silvia Masnada, Gaetan Lesca, Dorothée Ville, Patrizia Accorsi, Lucio Giordano, Anna Pichiecchio, Marialuisa Valente, Paola Borrelli, Ottavia Eleonora Ferraro, Pierangelo Veggiotti

**Affiliations:** ^1^ Department of Pediatric Neurology, Pediatric Neurology Unit Buzzi Children's Hospital Milan Italy; ^2^ Department of Genetics Lyon University Hospitals Lyon France; ^3^ Derpartment of Pediatric Neurology Lyon University Hospital Lyon France; ^4^ Child Neuropsychiatric Division Spedali Civili Brescia Italy; ^5^ Neuroradiology Department IRCCS C.Mondino National Neurological Institute Pavia Italy; ^6^ Clinical Pathology Unit, Medical Genetics Section SS. Annunziata Hospital Taranto Italy; ^7^ Department of Medical, Oral and Biotechnological Sciences, Laboratory of Biostatistics University “G. D'Annunzio” Pescara Italy; ^8^ Department of Public Health, Experimental and Forensic Medicine, Unit of Biostatistics and Clinical Epidemiology University of Pavia Pavia Italy; ^9^ Department of Biomedical and Clinical Sciences University of Milan Milan Italy

**Keywords:** ADSL deficiency, EEG patterns, epilepsy, genotype‐phenotype correlation, monogenic diseases

## Abstract

**Objective:**

Adenylosuccinate lyase (ADSL) deficiency is a rare inherited metabolic disorder with a wide phenotypic presentation, classically grouped into three types (neonatal, type I, and type II). We aim to better delineate the pathological spectrum, focusing on the electroclinical characteristics and phenotypic differences of patients with ADSL deficiency.

**Patients and Methods:**

Seven patients, from four different families, underwent serial electroencephalogram (EEG), clinical assessment, and neuroimaging. We also performed a systematic review of the cases published in the literature, summarizing the available clinical, neurophysiological, and genetic data.

**Results:**

We report seven previously unreported ADSL deficiency patients with long‐term follow‐up (10–34 years). From the literature review, we collected 81 previously reported cases. Of the included patient population, 58 % (51/88) were classified as having ADSL deficiency type I, 28% (25/88) as having type II, and 14% (12/88) as having neonatal. The most frequently reported pathogenic variants are p.R426H homozygous (19 patients), p.Y114H in compound heterozygosity (13 patients), and p.D430N homozygous (6 patients). In the majority (89.2%), disease onset was within the first year of life. Epilepsy is present in 81.8% of the patients, with polymorphic and often intractable seizures. EEG features seem to display common patterns and developmental trajectories: (i) poor general background organization with theta‐delta activity; (ii) hypsarrhythmia with spasms, usually adrenocorticotropic hormone‐responsive; (iii) generalized epileptic discharges with frontal or frontal temporal predominance; and (iv) epileptic discharge activation in sleep with an altered sleep structure. Imaging features present consistent findings of cerebral atrophy with frontal predominance, cerebellar atrophy, and white matter abnormalities among the three types.

**Significance:**

ADSL deficiency presents variable phenotypic expression, whose severity could be partially attributed to residual activity of the mutant protein. Although a precise phenotype‐genotype correlation was not yet feasible, we delineated a common pattern of clinical, neuroradiological, and neurophysiological features.

**Key Points**

- Adenylosuccinate lyase deficiency is a rare inherited metabolic disorder with a wide phenotypic presentation.
- Epilepsy is a core feature of the disease, with polymorphic and often drug‐resistant seizures.
- Patients display common electroencephalogram features and evolutionary trajectories.
- The severity of the phenotype seems to correlate with mutant protein residual activity.

## 1 INTRODUCTION

Adenylosuccinate lyase (ADSL) deficiency (OMIM 103050) is a rare autosomal recessive defect of the purine biosynthetic pathway caused by mutations in the *ADSL* gene, located on chromosome 22q13.1‐q13.2.^1^ From the first cases described by Jaeken and Van Den Berghe,^2^ currently, close to a 100 cases can be retrieved from the literature. ADSL is an enzyme that plays a key role in purine metabolism, catalyzing two non‐sequential steps: first, the conversion of succinylaminoimidazole carboxamide ribotide (SAICAr) to aminoimidazole carbozamide ribotide, and second, the conversion of adenylosuccinate (S‐AMP) to adenosine monophosphate (AMP). ADSL deficiency causes the buildup of by‐products, that is, SAICAr and S‐ADO, detectable in patients' CSF, urine, and plasma. The clinical presentation associated with ADSL deficiency can be extremely heterogeneous and, based on the age of onset and clinical severity, can be broadly classified into three main groups^1^:

- Type I or severe: most reported cases fall into this category. The onset is generally within the first months of life, and affected patients display the whole symptomatic spectrum of the disease, that is, severe developmental delay, epilepsy with intractable seizures, marked autistic features, axial hypotonia with limb hypertonia, dystonia, and ataxia.^3^
- Type II or mild: the onset of symptoms is generally within the first few years of life, but they can present later in life. Patients with type II ADSL deficiency generally present with mild to moderate developmental delay, autistic features, and variable pyramidal and extrapyramidal signs. They are less often characterized by seizures compared to the other groups.^4^
- Neonatal (N): The most severe form that presents at birth. Affected neonates are characterized by impaired intrauterine growth, microcephaly, fetal hypokinesia, a lack of heart rate variability, severe muscular hypotonia often leading to mechanical ventilation, resistant seizures, and early death.^5^

Epilepsy in ADSL deficiency is present in a consistent portion of the affected individuals.^1^ Seizures tend to display wide semiological variability and are often intractable, severely affecting the quality of life and the outcome of these patients.^6^ Here we describe seven previously unreported cases from four different families. We also performed a review of ADSL cases, attempting to establish possible genotype‐phenotype correlations and highlighting common clinical, neuroradiological, and neurophysiological features with specific reference to their epileptic background.

## 2 METHODS

We collected patients with a diagnosis of ADSL deficiency in two Italian centers (“Vittore Buzzi Children Hospital” in Milan and “ASST Spedali Civili” in Brescia) and one center in France (University Hospitals of Lyon). Clinical data were retrieved retrospectively from clinical registries and prospectively through interviews with patients, their families, and/or caregivers. Electroencephalographic (EEG) and video‐EEG recordings were available for all the patients at epilepsy onset and follow‐up. EEGs were obtained by a digital acquisition system, placing scalp electrodes according to the international 10–20 system. Selected patients underwent additional neurophysiologic investigations (ie, visual, auditory, and somatosensory evoked potentials and electroretinograms). All patients underwent sequential brain magnetic resonance (MRI), and the images were reviewed and discussed with a trained pediatric neuroradiologist. Five patients (Pt. 3–Pt. 7) underwent urine SAICAr and S‐Ado testing. All patients and their parents underwent genetic analysis using genomic DNA extracted from peripheral blood samples. Written informed consent was obtained from the parents or legal representatives of the involved patients.

### 2.1 Literature review

We performed a systematic review of the literature on ADSL deficiency cases. We searched different online repositories (PubMed, EMBASE, and Google Scholar) for all the relevant articles. The search terms included “*ADSL*”, “ADSL deficiency” and “Adenylosuccinate lyase deficiency”. All the articles were screened by title and abstract by two reviewers (GC and SM). We then hand‐searched relevant articles cited by the selected papers if they are not present in the initial search. All searches were carried out on October 10, 2022. We only included peer‐reviewed case reports or series published in peer‐reviewed journals in English, specifically reporting the genetic background and clinical features of the patients. The radiological data retrieved were reviewed by an expert child neuroradiologist. Patients without thorough clinical and/or genetic data were excluded from the synthesis (refer to Figure S1 for a flowchart of the screening process).

### 2.2 Statistical analysis

Descriptive analysis was carried out using the median and interquartile range (IQR) for the quantitative variables and percentage values for the qualitative ones. The normality distribution for quantitative variables was assessed by the Shapiro‐Wilk test. Pearson's chi‐square test or Fisher's exact test was used to evaluate the association between categorical variables, while the non‐parametric Kruskal–Wallis test was used to evaluate the differences between continuous variables and outcomes. After the Kruskal–Wallis test, for statistically significant results, the Dunn test was calculated for the comparison between the pairs of medians for the identification of significant differences. In addition, the survival analysis was performed by applying the Kaplan–Meier estimator and log‐rank test for equality of survivor functions. Statistical significance was set at the level of ≤0.05. All analyses were performed using Stata software v17.1 (StataCorp, College Station, USA).

## 3 RESULTS

### 3.1 Case series

Clinical data of the following patients are summarized in Table 1.

**TABLE 1:** Summary of the clinical features of reported cases.

	Family 1		Family 2		Family 3		Family 4
	Pt. 1	Pt. 2	Pt. 3	Pt. 4	Pt. 5	Pt. 6	Pt. 7
Sex, age, ethnicity	Male, dead at 19 years, Caucasian (Italian)	Male, 34 years, Caucasian (Italian)	Male, 22 years, Caucasian (Italian)	Female, 21 years, Caucasian (Italian)	Male, 26 years, Armenian	Female, 22 years, Armenian	Male, 23 years, Caucasian (Italian)
Mutation	c.1277G>A, p.R426H	c.1277G>A, p.R426H	c.1288G>A, p.D430N	c.1288G>A, p.D430N	c.1288G>A, p.D430N	c.1288G>A, p.D430N	p.Y114H; R296W
	(homozygous)	(homozygous)	(homozygous)	(homozygous)	(homozygous)	(homozygous)	(Compound heterozygosis)
Age disease onset	4 months	6 months	7 months	7 months	First year of age	First year of age	18 months
Presenting condition/s	Developmental delay with regression	Developmental delay with regression	Developmental delay	Hypotonia, developmental delay	Developmental delay	Developmental delay	Developmental delay
Developmental delay	Severe	Severe	Moderate	Moderate	Severe	Severe	Moderate
Cognition/language	Profound ID; no language	Profound ID; no language	Severe ID; only very few words	Moderate‐severe ID; words and short sentences	Severe ID; words and few short sentences	Moderate‐Severe ID; words and short sentences	Moderate‐severe ID; words and short sentences
Autistic features	Lack of interest in the environment and in social interactions, isolation, stereotypic hand movements	Lack of interest in the environment and in social interactions, isolation, stereotypic hand movements	Echolalia, stereotypic hand movements	Echolalia, hands, stereotypic movements	Lack of interest in the environment, stereotypic hand movement	No	Lack of interest in the environment and in social interactions; isolation; stereotypic hand movements
Additional neurological features	Axial hypotonia, limb hypertonia, pyramidal signs, extrapyramidal signs (distonic movements of the upper limbs), distal myoclonia, strabismus, nistagmus, macrocephaly, and tetraparesis	Axial hypotonia, limb hypertonia, pyramidal signs, extrapyramidal signs (facial grimaces), distal myoclonia, strabismus, nistagmus, and tetraparesis	Pyramidal signs (hypertonia and brisk reflexes), cerebellar signs (tremor, clumsiness, dysmetria, and ataxia), macrocephaly (>97°p)	Pyramidal signs and clumsiness	Nothing significative	Nothing significative	Limbs and axial hypotonia, microcephaly (<3°percentile), strabismus
Seizure onset	34 months	13 months	7 years	11 years	11 years	9 years	10 years
Seizure type	Spasms, focal seizures, FBTCS, GTCS, status epilepticus	Spasm, focal seizures, FBTCS, GTCS, status epilepticus	GTCS: myoclonic seizures of the head and upper limbs	GTS, GTCS, and FBTCS	GTCS	GTCS	GTCS, focal seizures
Seizure outcome	Never achieved seizure freedom	Daily GTCS and focal seizure	Seizure‐free	Seizure‐free	Seizure‐free	Seizure‐free	Seizure‐free
ASDs	BBX, PB, VPA, CZP, CBZ, NZP	**ACTH**, PB, VPA, NZP, GBP, TPM	**VPA, LEV**	**VPA**	CBZ, **VPA**	CBZ, **LTG, VPA**	**VPA**
EEG	Seizure onset: hypsarrhythmia	Seizure onset: hypsarrhythmia	Seizure onset: poor general organization	Seizure onset: poor general organization	Seizure onset: paroxysmal bilateral EDs	Seizure onset: diffuse spikes and Sp‐W	Seizure onset: diffuse spikes and Sp‐W predominantly in the fronto‐temporal regions
	Follow‐up: bilateral high‐voltage spikes and spikes and slow waves predominantly in fronto‐temporal regions.	Follow‐up: bilateral high‐voltage spikes and spikes and slow waves predominantly in fronto‐temporal regions.	Follow‐up: diffuse theta activity, EDs predominantly in the frontal temporal regions, generalized EDs	Follow‐up: poor general organization, low amplitude theta activity, EDs in centro‐temporal‐parietal regions, diffuse Sp‐Ws	Follow‐up: diffuse Sp‐Ws predominantly in the left frontal regions	Follow‐up: normal after VPA	Follow‐up: Poor general organization and EDs in fronto‐temporal regions
Additional neurophysiological examination	Visual and auditory evoked potentials and an electroretinogram showed a progressive deterioration	Visual and auditory evoked potentials and an electroretinogram showed a progressive deterioration	Auditory, visual, and somatosensory evoked potentials are normal	Auditory, visual, and somatosensory evoked potentials are normal	NA	NA	NA
MRI	8 months: normal	14 months: cerebral atrophy	15 months: normal	11 years: cerebellar vermis atrophy	17 years: diffuse cortical‐subcortical atrophy, predominantly in the hippocampal regions. White matter hyperintensities with an anterior predominance	13 years: diffuse cortical‐subcortical atrophy.	10 years: mild ventriculomegaly and periventricular white matter abnormalities
	10 years: progressive cerebral and cerebellar atrophy (frontal and parietal regions) and white matter hyperintensities (periventricular and posterior regions)	10 years and 19 years: progressive cerebral and cerebellar atrophy (frontal and parietal regions) and white matter hyperintensities (periventricular and posterior regions)	16 years: cerebellar vermis and cerebral atrophy, thin brainstem and corpus callosum, ventriculomegaly	17 years: progression of cerebellar vermis atrophy, cerebral atrophy, ventriculomegaly			
Additional features	Scoliosis, Naso‐Gastric tube feeding, hypogammaglobulinemia	Scoliosis, percutaneous gastrostomy, hypogammaglobulinemia	Scoliosis, multiple hypochromic skin lesions and “cafè au lait” macules, recurrent diarrhea, and recurrent abdominal pain	Multiple hypochromic skin lesions and “cafè au lait” macules, recurrent diarrhea, and recurrent abdominal pain	NA	NA	NA

*Note*: In the “ASDs” row the drug in bold is the one to which the patient responded better.

Abbreviations: ACTH, adrenocorticotropic hormone; ASDs, anti‐seizure drugs; BBX, barbexaclon; CZP, carbamazepine; EEG, electroencephalogram; ED, epileptic discharge; FBTCS, focal to bilateral tonic‐clonic seizure; GBP, gabapentin; GTCS, generalized tonic‐clonic seizure; GTS, generalized tonic seizure; ID, intellectual disability; LEV, levetirtacetam; LTG, lamotrigine; MRI, magnetic resonance imaging; mo, months; NZP, nitrazepam; PB, phenobarbital; Sp‐W, spike‐and‐wave; TPM, topiramate; VPA; valproic acid; y, years.

### 3.2 Family 1 (p.R426H, homozygous)

**FIGURE 1:**
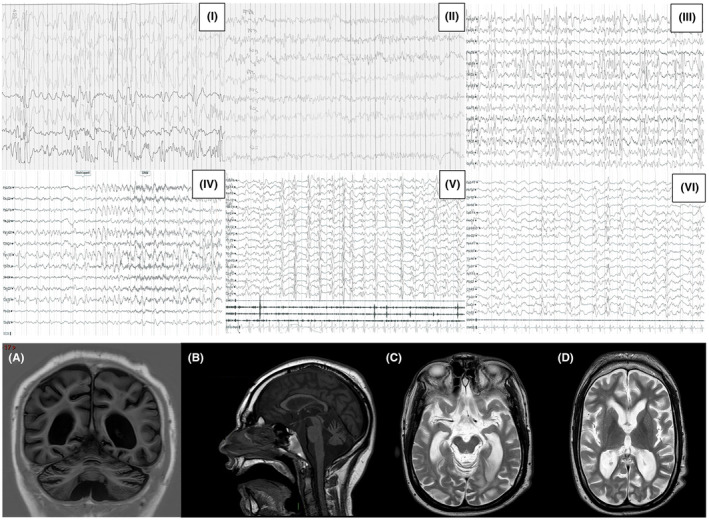
Electroencephalogram (EEG) and magnetic resonance imaging (MRI) evolution of Pt. 2. Panel I‐VI EEG of Pt. 2 from 12 months to 30 years of age (I) sleep EEG registration of Pt.2 (12 months year) shows hypsarrhythmia before the treatment with adrenocorticotropic hormone (ACTH); (II) awake EEG registration of Pt.2 after ACTH therapy shows a bilateral and symmetric 5–6 Hz background activity without epileptic discharges. (III) Awake EEG registration (13 years) showing high voltage, 1.5–2 Hz, spikes‐and‐waves predominantly over the frontal temporal regions. (IV) Awake EEG recording showing a disorganized background with spikes‐and‐waves predominantly on the frontal temporal regions and the start of a generalized seizure with clinical manifestations of staring spell and generalized stiffening. (V‐VI) Awake EEG (24 and 29 years) showing high voltage, bilateral spikes‐and‐spikes, and slow waves on frontal temporal regions only mildly reduced in frequency and amplitude over sequential controls. Pt. 2's MRI at 10 (A and B) and 19 years of age (C and D). (A) (inversion recovery, coronal plane) and B (T1, sagittal plane) show diffuse and generalized brain atrophy and ventriculomegaly with a thin and mildly dysmorphic corpus callosum. Cerebellar and vermian atrophy are also evident. Anterior commissure, optic nerves and olfactory bulbs were intact. (C and D) (T2, transversal plane) show a progression of brain atrophy, mainly in the frontal and temporal lobes (coherent with epileptic discharge localization), with areas of periventricular T2 hyperintensity. An analogous progression of EEG and MRI findings was documented for Pt.1.

Pt. 1 and Pt. 2 were born at term to non‐consanguineous Italian parents. Their prenatal and perinatal histories were unremarkable. At 4 and 6 months after birth, respectively, Pt. 1 and Pt. 2 presented with psychomotor regression of acquired motor and social skills, concomitant with the onset of epilepsy. Additionally, Pt. 1 exhibited strabismus and nystagmus. As they grew, both patients displayed profound intellectual disabilities and autistic features. Neurological examinations revealed spastic‐dystonic tetraparesis with axial hypotonia and four‐limb hypertonia. Extrapyramidal signs, such as upper limb dystonic movements with occasional non‐epileptic myoclonus, minimal head control, and macrocephaly (only observed in Pt. 1), were also noted. At onset, electroencephalographic evaluations showed a hypsarrhythmic pattern with epileptic spasms in both patients, leading to treatment with adrenocorticotropic hormone (ACTH), which achieved a few months of seizure control. In subsequent follow‐up EEGs up to adulthood, marked similarities between the two cases were observed, particularly the development of high‐voltage spikes and spike‐and‐slow waves predominantly in bilateral frontal and temporal regions. Epileptic discharges (EDs) activation during sleep, sometimes organized in bursts, were observed, leading to a progressive loss of sleep structure. Over time, focal seizures, focal to bilateral tonic‐clonic seizures, generalized tonic‐clonic seizures (GTCS), and several episodes of refractory status epilepticus were reported in both. Visual and auditory evoked potentials and electroretinogram showed a progressive deterioration over the years in the two brothers. Initial MRI scans for Pt. 1 and Pt. 2 (at 8 and 10 months of age, respectively) were normal. However, follow‐up MRI scans (at 10 years for Pt. 1, and 10 and 19 years for Pt. 2) revealed similar features: progressive cerebral and cerebellar atrophy, more pronounced in frontal and parietal regions, as well as ventriculomegaly, thin corpus callosum, brainstem abnormalities, and periventricular hyperintense T2 white matter signals. Genetic analysis through whole‐exome sequencing identified a homozygous pathogenic variant c.1277G>A (p.R426H) in the *ADSL* gene, inherited from healthy parents who are heterozygous carriers. Pt.1 died at 19 years of age of complications following a hospitalization for a respiratory tract infection. Pt. 2 is currently 34 years old and bedridden with daily generalized and focal seizures (refer to Figure 1 for a summary of EEG and MRI findings).

### 3.3 Family 2 (p.D430N, homozygous)

**FIGURE 2:**
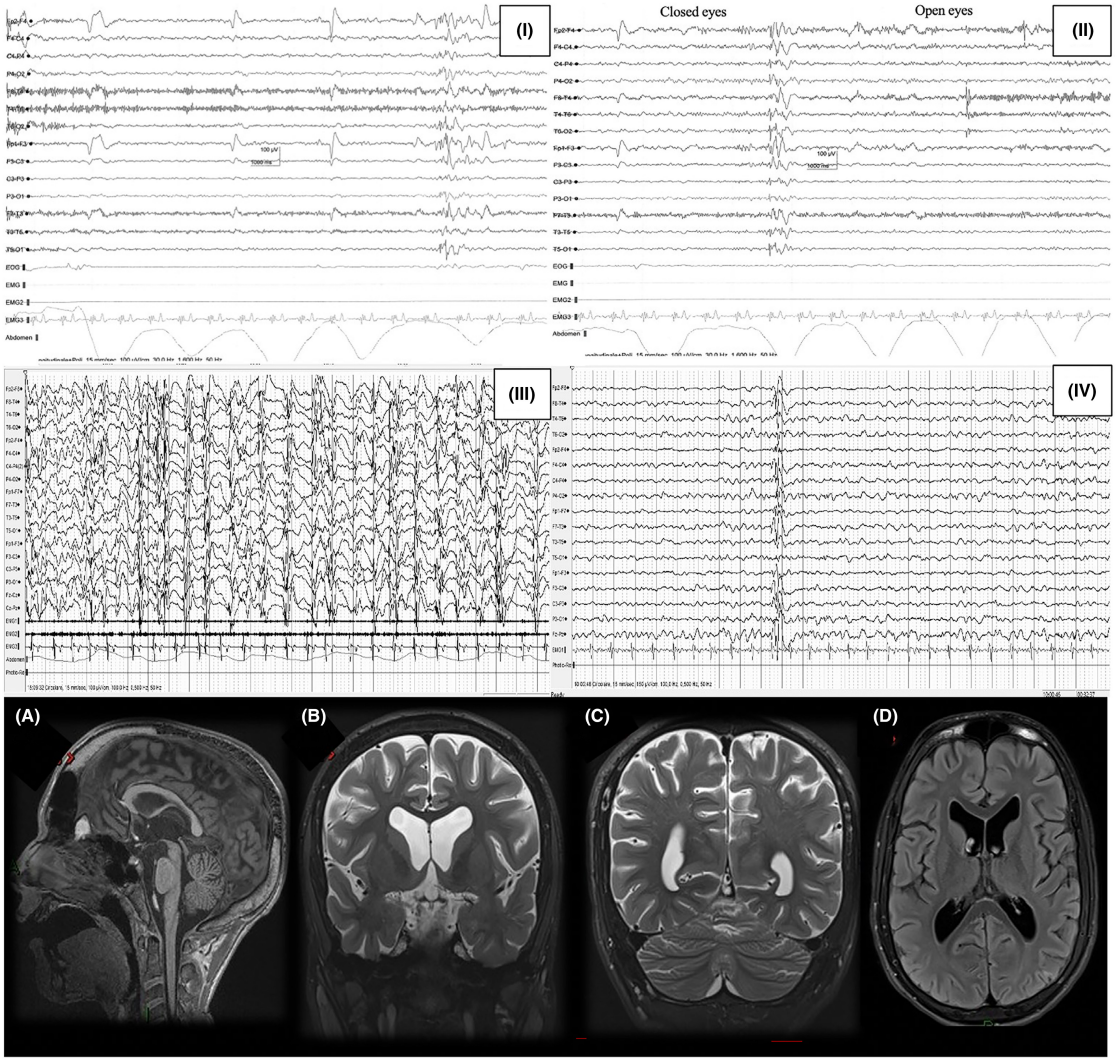
Electroencephalogram (EEG) and magnetic resonance imaging (MRI) of Pts. 3 and 4. Panels I and II show the EEG of Pt. 3 and Pt. 4, respectively (both at 16 years): they display a common pattern of poor general organization, prevalence of diffuse theta activity, poor reactivity to eye closure, and rare spikes or spikes‐and‐waves with frontal predominance; poor sleep organization without recognizable sleep phases was also observed. Panel III (Pt. 3, 12 years) shows an EEG during sleep with sequences of spike‐and‐wave activation in sleep evolving as the patient grew. Panel IV shows a sleep EEG of Pt.3 at 17 years in a dedifferentiation between awake and asleep states, with no recognizable sleep figures. Pt. 3 Brain MRI (16 years). (A) T1 sequence, sagittal plane: we can observe a thin corpus callosum and brainstem. Cerebellar atrophy is also noticeable. (B) T2 sequence, coronal plane: cerebral atrophy with enlarged ventricles. (C and D) T2 sequence, coronal and horizontal plane: marked cerebral and cerebellar atrophy are evident.

Pt. 3 and Pt. 4 are Caucasian siblings, born to non‐consanguineous Italian parents. They were born at term with unremarkable prenatal and perinatal histories. However, at around 7 months of age, both patients presented with developmental delay, which progressed to severe intellectual disability accompanied by prominent autistic features. It is worth noting that Pt. 4, the female patient, displayed a relatively milder phenotype compared to her brother, retaining better social and communicative skills. Upon neurological examinations, both patients exhibited brisk reflexes and limb hypertonia. Additionally, Pt. 3 displayed cerebellar signs such as tremor, dysmetria, and ataxia, as well as macrocephaly (>97 percentile). Both patients presented with multiple hypochromic skin lesions and “cafè au lait” macules, recurrent diarrhea, and abdominal pain. Seizures manifested in Pt. 3 and Pt. 4 at the ages of 7 and 11 years, respectively. EEG evaluations showed a loss of antero‐posterior gradient with spike‐and‐wave discharges predominantly localized over the frontal regions in both patients. In Pt. 4, diffuse epileptic discharges were also recorded. At the age of 12, Pt. 3 exhibited phases of continuous spike‐and‐wave activity during sleep, which progressively evolved to dedifferentiation of awake and asleep states. Treatment involved the use of valproic acid, with levetiracetam added for Pt. 3, and both achieved seizure freedom. However, no significant change in the EEG was observed. Brain MRI of the patients revealed similar features, namely, cerebral atrophy, mild periventricular leukoencephalopathy, and cerebellar atrophy (refer to Figure 2 for a summary of EEG and MRI findings). Whole‐exome sequencing analysis revealed that both patients carried a homozygous variant c.1288G>A (p.D430N) in the *ADSL* gene, which they inherited from their healthy parents. Interestingly, only Pt. 4 displayed elevated urinary SAICAr and S‐Ado levels. As of the last examinations, both patients remained seizure‐free under the administration of anti‐seizure medications (refer to Figure 2 for a summary of EEG and MRI findings).

### 3.4 Family 3 (p.D430N, homozygous)

Pt.5 and Pt.6 are French of Armenian descent. They presented with a psychomotor delay around the first year of life, more severe in Pt.5. Similar to previous cases, Pt. 6, the female patient, displayed a relatively milder phenotype compared to her brother. Pt. 6 did not exhibit overt autistic features, while Pt. 5 showed mild autistic traits. Neurological examinations were unremarkable except for the intellectual disability in both patients. Both developed epilepsy, respectively, at 11 and 9 years of age. The EEG showed diffused EDs more prominent in the frontal regions. Both patients achieved seizure freedom with valproate, associated with lamotrigine in Pt.6. Pt.6's EEG normalized after ASDs treatment. MRI showed generalized atrophy in both patients, with white matter periventricular T2 hyperintensities in Pt. 5. Only Pt. 5 showed elevated urinary SAICAr and S‐Ado. Whole genome analysis revealed the mutation c.1288G>A (p.D430N) in the *ADSL* gene, inherited from healthy parents. Currently, both patients are still seizure‐free under anti‐seizure drugs.

### 3.5 Family 4 (p.Y114H, p.R296W; compound heterozygosity)

Pt. 7, born to non‐consanguineous Italian parents, presented a relatively milder course of the disease. At around 18 months of age, the patient showed moderate psychomotor delay. Later, autistic behavior with motor stereotypies and limited social interaction became evident. Seizures developed at approximately 10 years of age, with both generalized and focal seizures observed. The EEG displayed a mild alteration of the general organization with frontal temporal spikes. The patient achieved seizure freedom with valproate monotherapy.

As of the last neurological examination, the patient displayed limb and axial hypotonia, along with strabismus. Brain MRI at the age of 10 showed mild periventricular leukoencephalopathy and ventriculomegaly, with no overt cerebral or cerebellar atrophy.

Elevated urinary levels of SAICAr and S‐Ado were observed in the patient. Whole‐exome analysis confirmed the diagnosis, revealing the presence of the variants p.Y114H and p.R296W of the *ADSL* gene. These variants were inherited from healthy parents. The patient is currently 23 years old and still seizure‐free with valproate monotherapy.

### 3.6 Review of the literature

Clinical characteristics and demographics are summarized in Tables 2 and 3, with 88 individuals in the patient population, 81 retrieved from 29 articles in the literature, and 7 newly described patients (Figure S1). Among the study population, 47.7% (42/88) were female. Globally, 58% (51/88) had ADSL deficiency type I, 28.4% (25/88) had type II, and 13.6% (12/88) had neonatal (type N) presentation. The age of disease onset varied: from birth in neonatal cases to 4 years: the majority (89.2%) within the first year of life, 29.5% within the first week, 15.9% between day 7 and day 30, 44.3% after the first month but within a year, and only 10.2% after the first year. Significant differences in median onset age were found between the groups (*P* < 0.0001). Specifically, type I versus type II (*P* < 0.0001), type I versus type N (*P* = 0.002), and type II versus type N (*P* < 0.0001). Epilepsy onset was earlier in type I and N patients compared to type II (*P* < 0.0001). The most common presenting symptom across disease types was developmental delay, observed in 47.7% (42/88) of patients, with varying severity across the disease spectrum, followed by seizures in 28.4% (25/88). Cardio‐respiratory deficits were seen in 10 patients, mostly in neonatal forms. Autistic features were reported in 56.9% (51/88) of patients, mainly in type I and type II, but sporadically in type N due to disease severity and early death. Hypotonia was present in 53 patients, more frequently in neonatal and type I groups (83% of neonatal form and 70.6% of type I ADSL deficiency, compared to 28% of milder variants). Pyramidal signs (spasticity and hyperreflexia) were in 33% (29/88) and extrapyramidal signs (dystonia and ataxia) in 12.5% (11/88), without specific group prevalence. Strabismus and ocular problems were in 26.1% (23/88) and microcephaly in 22.7% (20/88) of patients. Imaging data showed cerebral atrophy in 47.7% (42/88) of patients, most in type I (64%), and cerebellar atrophy, white matter abnormalities, and ventriculomegaly in significant proportions without specific group prevalence. Cerebral hemorrhages and gyrification deficits were mostly in the neonatal group. Data on EEG patterns and/or therapeutic strategies were collected and summarized in Table S1. From 16 articles specifically reporting on EEG features or therapeutic strategies, there were 26 patients. EEG features and localizations of the EDs varied considerably among different patients. EEG background was altered in all patients; hypsarrhythmia or a burst suppression pattern were reported in, respectively, four and six patients. Localization of the EDs varied and was reported as diffuse in five patients and focal in seven patients, mostly in the fronto‐temporal and more rarely in the occipital‐parietal regions. There were GTCS (17/26), followed by focal seizures (11/26), myoclonic seizures (11/26), spasms (5/26), and atypical absences (3/26). Valproic acid and levetiracetam were the most commonly reported ASDs used in these patients. Complete seizure control was reported in 5/26 patients.

**TABLE 2:** Demographics and clinical characteristics of the included patients (*N* = 88).

Gender, *n* (%)	
Female	42 (47.7%)
Male	46 (52.3%)
Type, *n* (%)	
I	51 (58.0%)
II	25 (28.4%)
N	12 (13.6%)
Age at presentation, *n* (%)	
<7 days	26 (29.5%)
8–30 days	14 (15.9%)
31–359	39 (44.3%)
≥360	9 (10.2%)
Presenting symptom, *n* (%)	
Seizure	25 (28.4%)
Developmental delay	42 (47.7%)
Hypotonia	14 (15.9%)
Cardiac and/or ventilatory disfunction	10 (11.4%)
Epilepsy, *n* (%)	72 (81.8%)
Seizure type, *n* (%)	
Generalized	33 (37.5%)
Focal	32 (36.4%)
Spasm	9 (10.2%)
Status	7 (8.0%)
Developmental delay, *n* (%)	82 (100%)
Degree, *n* (%)	
Mild	17 (25.0%)
Moderate	12 (17.6%)
Severe	39 (57.4%)
Autistic features, *n* (%)	51 (57.9%)
Hypotonia, *n* (%)	53 (60.2%)
Pyramidal signs, *n* (%)	29 (33.0%)
Extrapyramidal signs, *n* (%)	11 (12.5%)
Microcephaly, *n* (%)	20 (22.7%)
Respiratory system involvement, *n* (%)	17 (19.3%)
Cardiac disfunction, *n* (%)	6 (6.8%)
Strabismus/eye problems, *n* (%)	23 (26.1%)
Dysmorphism, *n* (%)	8 (9.1%)
Imaging features, *n* (%)	
Cerebral atrophy	42 (47.7%)
Cerebellar atrophy	15 (17.0%)
White matter abnormalities	36 (40.9%)
Ventriculomegaly/enlarged sulci	21 (23.9%)
Cerebral hemorrhage	5 (5.7%)
Gyrification deficits	4 (4.5%)

**TABLE 3:** Clinical characteristics and symptom prevalence according to disease type (ie, I, II, or N).

	Type (I, II, N)			
	I (*n* = 51)	II (*n* = 25)	N (*n* = 12)	*P*‐value
Age at presentation (days), median (IQR)	30.0 (7.0–150.0)	150.0 (120.0–360.0)	0.0 (0.0–2.5)	**<0.0001**
Epilepsy onset (days), median (IQR)	37.5 (13.5–375.0)	1800.0 (1440.0–3240.0)	0.0 (0.0–7.0)	**<0.0001**
Epilepsy, *n* (%)				
Not reported	7 (13.7%)	9 (36.0%)	0 (0.0%)	**0.014**
Present	44 (86.3%)	16 (64.0%)	12 (100.0%)	
Autistic features, *n* (%)				
Not reported	17 (33.3%)	9 (36.0%)	11 (91.7%)	**0.001**
Present	34 (66.7%)	16 (64.0%)	1 (8.3%)	
Aggressive behavior, *n* (%)				
Not reported	41 (80.4%)	21 (84.0%)	12 (100.0%)	0.292
Present	10 (19.6%)	4 (16.0%)	0 (0.0%)	
Hypotonia, *n* (%)				
Not reported	15 (29.4%)	18 (72.0%)	2 (16.7%)	**<0.0001**
Present	36 (70.6%)	7 (28.0%)	10 (83.3%)	
Pyramidal signs, *n* (%)				
Not reported	30 (58.8%)	18 (72.0%)	11 (91.7%)	0.082
Present	21 (41.2%)	7 (28.0%)	1 (8.3%)	
Extrapyramidal signs, *n* (%)				
Not reported	45 (88.2%)	20 (80.0%)	12 (100.0%)	0.279
Present	6 (11.8%)	5 (20.0%)	0 (0.0%)	
Microcephaly, *n* (%)				
Not reported	37 (72.5%)	21 (84.0%)	10 (83.3%)	0.551
Present	14 (27.5%)	4 (16.0%)	2 (16.7%)	
Respiratory symptoms, *n* (%)				
Not reported	44 (86.3%)	25 (100.0%)	2 (16.7%)	**<0.0001**
Present	7 (13.7%)	0 (0.0%)	10 (83.3%)	
Cardiac symptoms, *n* (%)				
Not reported	51 (100.0%)	25 (100.0%)	6 (50.0%)	**<0.0001**
Present	0 (0.0%)	0 (0.0%)	6 (50.0%)	
Strabismus/eye problems, *n* (%)				
Not reported	34 (66.7%)	19 (76.0%)	12 (100.0%)	**0.045**
Present	17 (33.3%)	6 (24.0%)	0 (0.0%)	
Dysmorphism, *n* (%)				
Not reported	47 (92.2%)	23 (92.0%)	10 (83.3%)	0.509
Present	4 (7.8%)	2 (8.0%)	2 (16.7%)	
Cerebral atrophy, *n* (%)				
Not reported	18 (35.3%)	17 (68.0%)	11 (91.7%)	**<0.0001**
Present	33 (64.7%)	8 (32.0%)	1 (8.3%)	
Cerebellar atrophy, *n* (%)				
Not reported	39 (76.5%)	22 (88.0%)	12 (100.0%)	0.125
Present	12 (23.5%)	3 (12.0%)	0 (0.0%)	
White matter abnormalities, *n* (%)				
Not reported	26 (51.0%)	17 (68.0%)	9 (75.0%)	0.209
Present	25 (49.0%)	8 (32.0%)	3 (25.0%)	
Ventriculomegaly/enlarged sulci, *n* (%)				
Not reported	38 (74.5%)	18 (72.0%)	11 (91.7%)	0.434
Present	13 (25.5%)	7 (28.0%)	1 (8.3%)	
Cerebral hemorrhage, *n* (%)				
Not reported	50 (98.0%)	25 (100.0%)	8 (66.7%)	**0.001**
Present	1 (2.0%)	0 (0.0%)	4 (33.3%)	
Gyrification deficits, *n* (%)				
Not reported	50 (98.0%)	25 (100.0%)	9 (75.0%)	**0.007**
Present	1 (2.0%)	0 (0.0%)	3 (25.0%)	

*Note*: The values in bold are the statistically significant one.

### 3.7 Focus on genotype‐phenotype correlation

The most frequently reported variants in our sample are: (i) the homozygous p.R426H variant found in 19 patients; (ii) the p.Y114H variant reported in compound heterozygosity in 13 patients (4 of them presented the variant in conjunction with the p.R426H); and (iii) the variants p.D430N reported in 6 patients (4 homozygous and 2 heterozygous with the p.R426H variant). Most variants in the literature were inherited from healthy carrier parents. Kmoch et al. categorized seven ADSL variants based on their residual activities: null variants without detectable activity (p.Y114H and p.D268N), variants with substantially compromised activity (p.R194C and p.R426H), and variants with activities similar to the wild‐type enzyme (p.A3V, p.R190Q, and p.D430N).^7^ Patients with combined p.Y114H and p.R426H variants showed the most severe phenotype. Mouchegh et al.^5^ reported four cases with p.Y114H and p.R426H variants in compound heterozygosis, presenting severe cardio‐respiratory complications and refractory seizures immediately after birth. Other patients with at least one p.Y114H allele also showed early presentation with a similar clinical picture, though some had milder phenotypes when p.Y114H was present in compound heterozygosity, for example, Pts. 4 and 5 in Kmoch et al.^7^ whose other allele was p.R190Q, had a relatively milder phenotype without cardio‐respiratory complications or epilepsy. Three patients with p.Y114H and p.G418A were reported (Pts. 6–8 in Mastrogiorgio et al.^8^), two with type II and one with type I, all having mild developmental delay, and two with epilepsy. Homozygous p.R426H carriers reported in the literature, like our Pts. 1 and 2, presented early with developmental delay, sometimes associated with regression of acquired skills, drug‐resistant epilepsy, and often with spastic‐dystonic tetraparesis. An exception is seen in five cases harboring the same homozygous variant (Pt. 1 and Pt. 2 in Marie et al.^9^; Pt. 3 in Edery et al.^10^; Pt. 5 in Donti et al.^3^; Pt. 5 in Mastrogiorgio et al.^8^) where no epilepsy was reported, suggesting the involvement of unaccounted gene‐gene interactions or non‐genetic factors in the phenotype. The p.D430N variant encodes a mutant protein with modest residual function and was reported in two patients (Pt. 1 in Jurecka et al.^4^ and Pt.16 in Mastrogiorgio et al.^8^) in heterozygosity with p.R426H, resulting in a mild phenotype without epilepsy. Our patients with homozygous p.D430N (Pts. 2–6) had a milder phenotype with developmental delay and autistic features within the first year and epilepsy onset in late childhood, achieving seizure freedom with appropriate treatment. The newly reported p.P24L variant's protein activity is not yet characterized. The four patients with p.P24L variants are compound heterozygous, and their phenotype, while consistent in terms of developmental delay and epilepsy presence, displays different degrees of severity even between siblings.^11^ Only a few patients were reported with additional variants, severely limiting the description of peculiar traits related to specific protein changes.

### 3.8 Survival analysis

**FIGURE 3:**
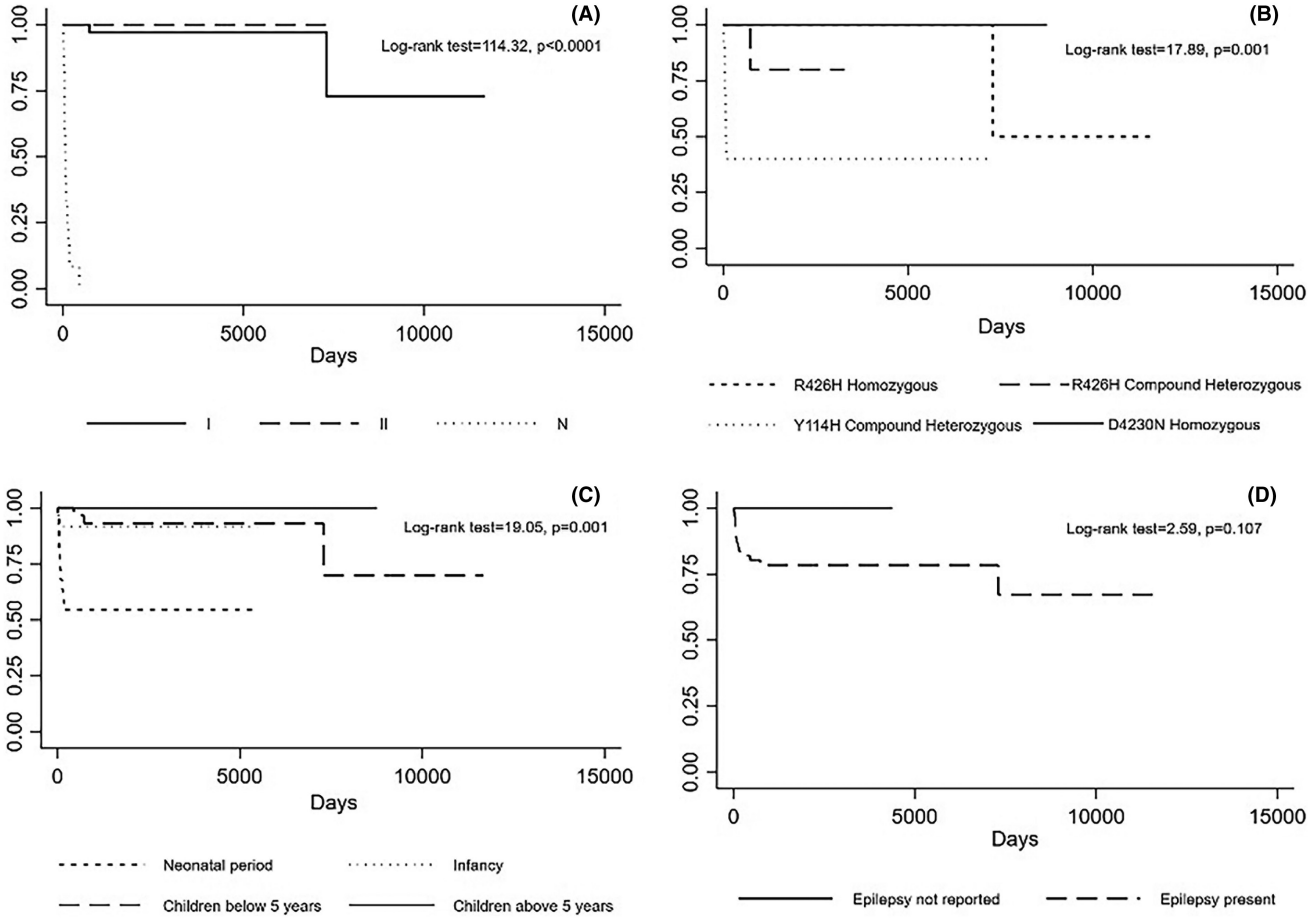
Kaplan–Meier curves estimating survivals in different groups of patients. (A) Compares the different forms of the disease, showing lower survival rates in type N and I compared to type II patients. (B) Compares the most frequent variants observed in the sample, highlighting how lower survival is observed in p.Y114H heterozygous and p.R426H homozygous carriers. (C) Compares survival based on the age of onset, displaying how an earlier onset, not accounting for mutation type, was also associated with a poorer prognosis. (D) Compares the population of patients with epilepsy and the population without showing any statistically significant difference in terms of survival between the two population; however, we can notice a trend toward lower survival for patients with epilepsy.

As expected, neonatal forms are associated with lower survival compared to the other two groups (Figure 3A). We computed the survival of the carriers of the four most frequent variants, that is, homozygous p.R426H, compound heterozygous p.Y114H, compound heterozygous p.R426H (excluding the p.Y114H and p.R426H carriers computed in the previous group), and homozygous p.D430N. The analysis revealed poor survival in the compound heterozygous p.Y114H and p.R426H groups and a better prognosis of the homozygous p.R426H and p.D430N carriers (*Panel B*, log‐rank test = 17.89, *P* = 0.001). The neonatal onset, not accounting for mutation type, was associated with a poorer prognosis (*Panel C*). The presence of epilepsy is not associated with a statistically significant difference in terms of survival (*Panel D*). However, an earlier onset of epilepsy correlated with a worse prognosis (log‐rank test = 8.92, *P* = 0.030). Seizures as the presenting condition and white matter abnormalities did not show significant differences in terms of survival (log‐rank test = 0.37, *P* = 0.540; log‐rank test = 1.07, *P* = 0.300, respectively).

## 4 DISCUSSION

Although ADSL deficiency is classified into three broad phenotypes, the condition remains highly heterogeneous. The mechanisms by which ADSL deficiency can give rise to its symptomatology are not fully elucidated and may include deficiency of purine nucleotides, impairment of cellular bioenergetics, and toxic accumulation of SAICAr and S‐ADO.^6^ Despite a diverse clinical presentation, epilepsy is present in a substantial portion of the affected patients (ie, 81.8%), with polymorphic and often intractable seizures, severely affecting the quality of life of the patients and their caregivers. Earlier epilepsy onset and drug‐resistant seizures appear to be more frequent in patients with type I and neonatal phenotypes and in patients harboring pathogenic variants encoding proteins with reduced residual activity. Different EEG patterns have been observed in ADSL patients: (i) a poor background organization with diffuse theta‐delta activity was commonly reported; (ii) a burst suppression pattern or hypsarrhythmic pattern with epileptic spasms, especially at onset, has been described. As reported in our patients and by other authors,^12^, ^13^ the hypsarrhythmic pattern is generally responsive to ACTH or steroidal therapy, although the response is only limited in time. Also, burst suppression patterns have been reported. (iii) These patterns may evolve during childhood or adolescence into focal spike‐and‐waves with a predominance over the frontal temporal regions of EDs, as observed in our two first siblings and in previous data from the literature.^14^, ^15^, ^16^ Despite the wide clinical spectrum, the frontal or fronto‐temporal predominance of EDs observed in all our cases and reported in previous literature,^14^, ^15^, ^16^ could be considered a possible specific localization of EDs in this condition and seems to coincide with the areas that show the most significant atrophy on MRI.^17^ Sporadically, occipital predominance or diffused spike‐and‐waves have also been observed,^15^, ^18^ (iv) sleep patterns were rarely reported,^19^ and like our patients, the sleep structure was disorganized with no recognizable physiological sleep elements. No overt activation of spike‐and‐wave activity in sleep was previously reported; however, sleep recordings of our Pt. 3 and, to a lesser extent, Pts.1 and 2, showed EDs activation, also in bursts, evolving to a progressive impoverishment of the EEG background with loss of physiological sleep elements. EDs sleep activation in our Pt. 3, despite not configuring a typical case of developmental and/or epileptic encephalopathy with spike‐and‐wave activation in sleep (DEE‐SWAS) due to a different clinical evolution and genetic background, may account for his more severe cognitive phenotype in comparison with the sibling. Concerning the anti‐seizure treatment for ADSL deficiency, to date, no standard of care can be recommended. However, in our milder patients (Pts. 3–7), valproate, levetiracetam, and lamotrigine were found to be effective. Ketogenic diet, D‐ribose, and S‐adenosyl‐L‐ methionine^1^, ^11^, ^12^, ^17^, ^20^ were implemented as possible therapeutic strategies in these patients with limited success (refer to Table S2). Clinical features, for example, psychomotor delay, intellectual disability, epilepsy, frequently associated with autistic traits, pyramidal and extrapyramidal signs, albeit common to many childhood encephalopathies, can be suggestive of this condition and configure a spectrum of disease presentation with differences in severity among the types of the disease.

Also, imaging can aid in suspecting of ADSL deficiency, with similar features but with different grade of severity among the different phenotypes: cerebral, cerebellar atrophy, and white matter abnormalities tend to be more pronounced in Pts.1 and 2 (type I) compared to the patients we have described in families 2 and 3 (type II). These data were confirmed by the literature, where cerebral and cerebellar atrophy were reported in a significant proportion of patients, as well as white matter abnormalities (ie, periventricular or semioval center T2‐hyperintensities). A previous review^17^ specifically focusing on the MRI features of ADSL patients reported cerebral atrophy with frontal predominance as a common finding in older patients, in addition to cerebellar, specifically vermian, atrophy and white matter periventricular abnormalities. Such findings, in our cases and in literature,^17^ tend to be more prominent in the most severe types of ADLS deficiency (type I) compared to the milder (type II) and more evident in older patients. They are not often reported in neonatal forms, in which cortical development abnormalities or cerebral hemorrhages are most commonly encountered, contributing to their poor prognosis and early death (preventing the establishment of overt cerebral atrophy).

Considering the features described in our new patients reported, in the literature revision, and the previous functional analysis conducted, the phenotype of the patients affected by ADSL deficiency seems to suggest a possible correlation with the residual activity of the ADSL protein.^7^ Patients harboring the p.Y114H variant, with minimal residual function, presented with neonatal forms of the disease, while patients with variants encoding for proteins with an almost normal residual function (eg, p.R190Q and p.D430N) presented with a relatively milder course. However, rarely, compound heterozygous patients can show a milder phenotype, for example, our Pt. 7 (p.Y114H and p.R296W), suggesting the influence of the residual activity of the mutant protein encoded in the second allele in the establishment of the phenotype. Patients with the homozygous p.R426H variants, like our first family described, generally presented with a type I phenotype even if considerable variability could be observed between affected individuals, while patients with variants encoding for proteins with an almost normal residual function (eg, p.R190Q and p.D430N, like our reported patients) presented with a relatively milder course. Functional studies on new variants and the identification of new patients would be required to delineate a stronger phenotype‐genotype correlation.

### 4.1 Limitations

In our review, we included only a part of the literature published on ADSL patients because we focused only on peer‐reviewed English‐language journals and we included only studies or clinical cases reporting both genetic and clinical data. To date, many variants are present only in small groups of patients and are not fully clinically characterized. For a database of the variants identified up to June 2013, please refer to http://www1.lf1.cuni.cz/udmp/ADSL/. The low number of people with the disease and the high variability between the reported variants did not allow for a cluster analysis of the symptoms based on specific protein alterations. This analysis will be the goal of future work expanding the sample.

## 5 CLINICAL RELEVANCE AND FUTURE DIRECTIONS

We present a series of previously unreported patients with ADSL deficiency with long‐term follow‐up documenting the electroclinical features of the syndrome and comparing our patients with previously published cases. Despite the fact that a precise genotype‐phenotype association is not feasible due to the limited number of patients reported, clinical phenotype severity seems to correlate with residual protein activity. Also, clinical, neuroradiological, and neurophysiological data seem to display common features and developmental trajectories in ADSL patients: the development of cerebral and cerebellar atrophy and white matter periventricular abnormalities associated with an EEG pattern of EDs with frontal temporal predominance. In patients with psychomotor delay, epilepsy, prominent autistic features, and pyramidal‐extrapyramidal signs, ADSL deficiency might be considered in the differential diagnosis of epileptic encephalopathies.

## CONFLICT OF INTEREST STATEMENT

The authors have no disclosures related to the present work.

## ETHICS STATEMENT

Written informed consent was obtained from the parents or legal representatives of the involved patients. The study adheres to the principles of the Code of Ethics of the World Medical Association‐Helsinki Declaration and concerns data gathered during routine diagnostic activity. The study also complies with institutional regulations for anonymized retrospective studies. We confirm that we have read the Journal's position on issues involved in ethical publication and affirm that this report is consistent with those guidelines.

## Supporting information


Figure S1:
Table S1:

## Data Availability

Data to support the findings of this study are included in the article and supplementary materials. Additional data may be available from the corresponding author, PV, upon reasonable request.
