# Supplementary material for: Electroclinical features and phenotypic differences in adenylosuccinate lyase deficiency: Long‐term follow‐up of seven patients from four families and appraisal of the literature
Source: Epilepsia Open. 2023 Nov 27;9(1):106–21. doi: 10.1002/epi4.12837 (PMC10839293; doi:10.1002/epi4.12837)
Supplement: Supplementary file 1 — Figure S1: Table S1: [file EPI4-9-106-s001.zip › Cutillo et al.Supplementary material.docx]

**Electroclinical features and phenotypic differences in Adenylosuccinate lyase deficiency: long term follow-up of seven patients from four families and appraisal of the literature - Supplementary material**

Records removed *before screening*:

Duplicate records removed

(n = 230)

Records identified from:

Pubmed (n = 265)

Embase (n = 362)

Google Scholar (hand searched, no additional results)

**Identification**

Inclusion criteria

- English language
- Peer-reviewed
- Available clinical information
- Available genetic data

Records screened by title and abstract

(n = 397)

**Screening**

Full text screening (n = 97)

Excluded articles (n = 68)

Studies included in review

(n = 29)

Patient in the included studies

(n = 88)

**Included**

***Figure S1:*** Flowchart of the screening process (Adapted from Page MJ, McKenzie JE, Bossuyt PM, Boutron I, Hoffmann TC, Mulrow CD, et al. The PRISMA 2020 statement: an updated guideline for reporting systematic reviews. BMJ 2021;372:n71. doi: 10.1136/bmj.n71).

**
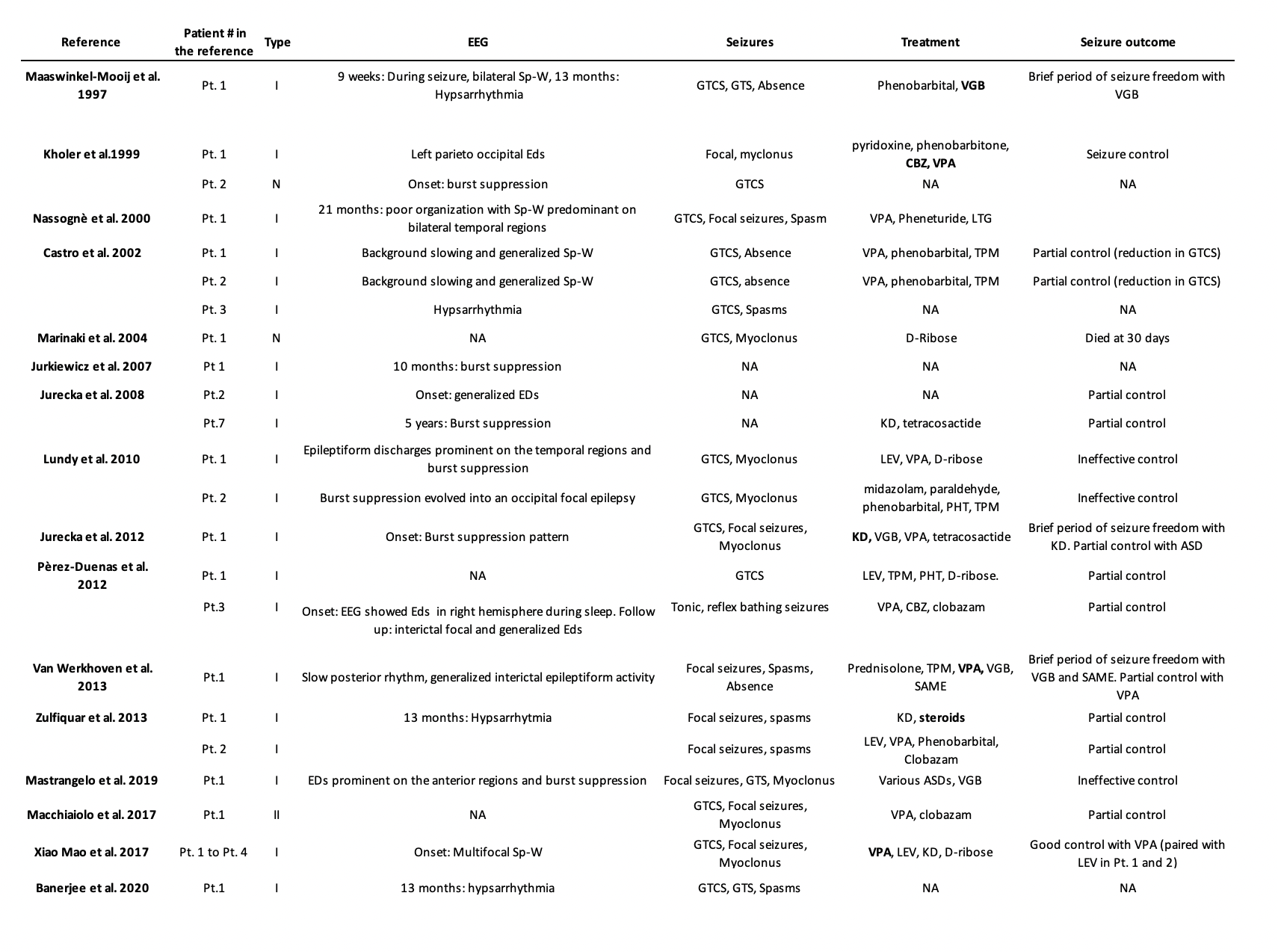
**

***Table S1:*** *Summary of the articles specifically reporting on electroencephalographic and/or epileptological features of the patients Abbreviations: ASDs: Anti-seizure drugs; CZP: carbamazepine; ED: epileptic discharge; GTCS: Generalized tonic-clonic seizure; GTS: generalized tonic seizure; KD: ketogenic diet; LEV: levetiracetam; LTG: lamotrigine; SAME: S-Adenosyl methionine; Sp-W: spike-and-wave; TPM: topiramate; VGB: vigabatrin, VPA: Valproic acid. In the “treatment” row the drug in bold is the one to which the patient responded better.*
