# Supplementary material for: Electroclinical features and phenotypic differences in adenylosuccinate lyase deficiency: Long‐term follow‐up of seven patients from four families and appraisal of the literature
Source: Epilepsia Open. 2023 Nov 27;9(1):106–21. doi: 10.1002/epi4.12837 (PMC10839293; doi:10.1002/epi4.12837)
Supplement: Supplementary file 1 — Figure S1: Table S1: [file EPI4-9-106-s001.zip › Table S2 ADSL.docx]

| **Reference** | **Patient # in the reference** | **Type** | **EEG** | **Seizures** | **Treatment** | **Seizure outcome** |
| --- | --- | --- | --- | --- | --- | --- |
| **﻿Maaswinkel-Mooij et al. 1997** | Pt. 1 | I | 9 weeks: During seizure, bilateral Sp-W, 13 months: Hypsarrhythmia | GTCS, GTS, Absence | Phenobarbital, **VGB** | Brief period of seizure freedom with VGB |
| **Kholer et al.1999** | Pt. 1 | I | Left parieto occipital Eds | Focal, myclonus | pyridoxine, phenobarbitone, **CBZ, VPA** | Seizure control |
|  | Pt. 2 | N | Onset: burst suppression | GTCS | NA | NA |
| **Nassognè et al. 2000** | Pt. 1 | I | 21 months: poor organization with Sp-W predominant on bilateral temporal regions | GTCS, Focal seizures, Spasm | VPA, Pheneturide, LTG |  |
| **Castro et al. 2002** | Pt. 1 | I | Background slowing and generalized Sp-W | GTCS, Absence | VPA, phenobarbital, TPM | Partial control (reduction in GTCS) |
|  | Pt. 2 | I | Background slowing and generalized Sp-W | GTCS, absence | VPA, phenobarbital, TPM | Partial control (reduction in GTCS) |
|  | Pt. 3 | I | Hypsarrhythmia | GTCS, Spasms | NA | NA |
| **Marinaki et al. 2004** | Pt. 1 | N | NA | GTCS, Myoclonus | D-Ribose | Died at 30 days |
| **Jurkiewicz et al. 2007** | Pt 1 | I | 10 months: burst suppression | NA | NA | NA |
| **Jurecka et al. 2008** | Pt.2 | I | Onset: generalized EDs | NA | NA | Partial control |
|  | Pt.7 | I | 5 years: Burst suppression | NA | KD, tetracosactide | Partial control |
| **Lundy et al. 2010** | Pt. 1 | I | Epileptiform discharges prominent on the temporal regions and burst suppression | GTCS, Myoclonus | LEV, VPA, D-ribose | Ineffective control |
|  | Pt. 2 | I | Burst suppression evolved into an occipital focal epilepsy | GTCS, Myoclonus | midazolam, paraldehyde, phenobarbital, PHT, TPM | Ineffective control |
| **Jurecka et al. 2012** | Pt. 1 | I | Onset: Burst suppression pattern | GTCS, Focal seizures, Myoclonus | **KD,** VGB, VPA, tetracosactide | Brief period of seizure freedom with KD. Partial control with ASD |
| **Pèrez-Duenas et al. 2012** | Pt. 1 | I | NA | GTCS | LEV, TPM, PHT, D-ribose. | Partial control |
|  | Pt.3 | I | Onset: EEG showed Eds in right hemisphere during sleep. Follow up: interictal focal and generalized Eds | Tonic, reflex bathing seizures | VPA, CBZ, clobazam | Partial control |
|  |  |  |  |  |  |  |
| **Van Werkhoven et al. 2013** | Pt.1 | I | Slow posterior rhythm, generalized interictal epileptiform activity | Focal seizures, Spasms, Absence | Prednisolone, TPM, **VPA,** VGB, SAME | Brief period of seizure freedom with VGB and SAME. Partial control with VPA |
| **Zulfiquar et al. 2013** | Pt. 1 | I | 13 months: Hypsarrhytmia | Focal seizures, spasms | KD, **steroids** | Partial control |
|  | Pt. 2 | I |  | Focal seizures, spasms | LEV, VPA, Phenobarbital, Clobazam | Partial control |
| **Mastrangelo et al. 2019** | Pt.1 | I | EDs prominent on the anterior regions and burst suppression | Focal seizures, GTS, Myoclonus | Various ASDs, VGB | Ineffective control |
| **Macchiaiolo et al. 2017** | Pt.1 | II | NA | GTCS, Focal seizures, Myoclonus | VPA, clobazam | Partial control |
| **Xiao Mao et al. 2017** | Pt. 1 to Pt. 4 | I | Onset: Multifocal Sp-W | GTCS, Focal seizures, Myoclonus | **VPA**, LEV, KD, D-ribose | Good control with VPA (paired with LEV in Pt. 1 and 2) |
| **Banerjee et al. 2020** | Pt.1 | I | 13 months: hypsarrhythmia | GTCS, GTS, Spasms | NA | NA |

***Table S2:*** *Summary of the articles specifically reporting on electroencephalographic and/or epileptological features of the patients Abbreviations: ASDs: Anti-seizure drugs; CZP: carbamazepine; ED: epileptic discharge; GTCS: Generalized tonic-clonic seizure; GTS: generalized tonic seizure; LEV: levetiracetam; Sp-W: spike-and-wave; TPM: topiramate; VPA: Valproic acid. In the “treatment” row the drug in bold is the one to which the patient responded better.*
